# gmcoda: Graphical model for multiple compositional vectors in microbiome studies

**DOI:** 10.1093/bioinformatics/btad700

**Published:** 2023-11-17

**Authors:** Huaying Fang

**Affiliations:** Beijing Advanced Innovation Center for Imaging Theory and Technology, Capital Normal University, Beijing 100048, China; Academy of Multidisciplinary Studies, Capital Normal University, Beijing 100048, China

## Abstract

**Motivation:**

Microbes are essential components in the ecosystem and participate in most biological procedures in environments. The high-throughput sequencing technologies help researchers directly quantify the abundance of microbes in a natural environment. Microbiome studies explore the construction, stability, and function of microbial communities with the aid of sequencing technology. However, sequencing technologies only provide relative abundances of microbes, and this kind of data is called compositional data in statistics. The constraint of the constant-sum requires flexible statistical methods for analyzing microbiome data. Current statistical analysis of compositional data mainly focuses on one compositional vector such as bacterial communities. The fungi are also an important component in microbial communities and are always measured by sequencing internal transcribed spacer instead of 16S rRNA genes for bacteria. The different sequencing methods between fungi and bacteria bring two compositional vectors in microbiome studies.

**Results:**

We propose a novel statistical method, called gmcoda, based on an additive logistic normal distribution for estimating the partial correlation matrix for cross-domain interactions. A majorization–minimization algorithm is proposed to solve the optimization problem involved in gmcoda. Through simulation studies, gmcoda is demonstrated to work well in estimating partial correlations between two compositional vectors. Gmcoda is also applied to infer cross-domain interactions in a real microbiome dataset and finds potential interactions between bacteria and fungi.

**Availability and implementation:**

Gmcoda is open source and freely available from https://github.com/huayingfang/gmcoda under GNU LGPL v3.

## 1 Introduction

Microbes are small organisms living almost everywhere in natural environments, and most of them cannot be observed without the help of a microscope. Microbes influence their surroundings and vice versa. For example, some bacterial species in the gut are suggested to interact with host metabolism and influence host phenotypes such as obesity and diabetes (Patterson *et al.* 2016). Microbes do not exist solely, and they interact with each other to maintain the stability of the micro-ecosystem where they live. Microbiome studies explore and validate the structure and function of microbes including bacteria and fungi in the natural micro-ecosystem while abundances of microbes are measured by high-throughput sequencing technologies.

However, the microbiome experiment can only provide relative abundances, which is also called compositional data. Two main characteristics of compositional data are that components are non-negative, and the sum of all components is constant. This constant-sum constraint for compositional data brings difficulties in analyzing the interaction network by using classical methods. For example, Pearson correlations of components have a negative trend of correlations between components. Another challenge in microbiome data is that the sample size is much smaller than the number of microbial species in microbiome studies. The compositional and high-dimensional features of microbiome data provide new challenges and opportunities for statisticians and data scientists.

There are a variety of methods for inferring correlation structure among microbes with the compositional effect in microbiome studies (Friedman and Alm 2012, Fang *et al.* 2015, Cao *et al.* 2019). The pairwise correlations do not consider the impact of other microbes for estimating the interaction between two microbes. In contrast, the partial correlation matrix in the Gaussian graphical model can be used to measure the conditional dependence between two random variables given others, such that an element in the partial correlation matrix is 0 if and only if the two microbial species are conditionally independent given all other species (Yuan and Lin 2007). Kurtz *et al.* (2015) proposed an approximate procedure, named SPIEC-EASI (SParse InversE Covariance estimation for Ecological ASsociation Inference), to infer the partial correlation among components by the sparse neighborhood or the inverse covariance selection, but this approximation may fail in some situations. Fang *et al.* (2017) extended the Gaussian graphical model in compositional data by an additive logistic normal distribution.

To date, a majority of microbiome analyses measure the relative abundance of bacteria using the 16S RNA gene as the marker gene. The resulting data consists of a single compositional vector. Most existing graphical models for compositional data have also been designed to model this kind of compositional vector data. Increasingly, however, multiple compositional vectors have arisen in microbiome studies. As an example, fungi are important in maintaining the stability of the micro-ecosystem. The relative abundance of fungi species can be quantified based on the internal transcribed spacer (ITS), in much a similar fashion as the 16S rRNA gene for bacteria. Thus, the same biospecimen and the same sequencing run can produce two or more compositional vectors describing microbial communities: one for bacteria and another for fungi. Furthermore, multiple compositional vectors can also appear for different body sites of 16S rRNA genes in the same subject. Lastly, some recent microbiome studies have included, as environmental variables, compositional vectors representing mass-spectrometry generated metabolites. The metabolite abundances quantified from mass spectrometry are also relative data. Most existing methods do not allow for jointly modeling multiple compositional data. Tipton *et al.* (2018) applied an extension version of SPIEC-EASI for exploring the partial correlation between bacteria and fungi in microbial studies, and this extension version also inherits the drawback of SPIEC-EASI that the approximation may be not good in some cases.

In this article, we propose a novel statistical method called gmcoda for inferring the partial correlation matrix of microbes from multiple compositional vectors based on the additive logistic normal distribution. The additive logistic normal distribution is the extension of the model proposed in Fang *et al.* (2015, 2017). This model assumes that the observed compositional data are from an additive logistic transformation of latent variables and the total abundances of compositional vectors are lost in the transformation. We derive the likelihood function under the multivariate normal assumption of the latent variables and propose a penalized likelihood method to estimate a sparse partial correlation matrix for multiple compositional vectors. A majorization–minimization algorithm is proposed to solve the optimization problem in the penalized likelihood. Simulation studies and real data analysis are performed to show the advantages of the proposed method. Applied to a real dataset, we demonstrate that the proposed method detects a large number of interactions between bacteria and fungi. The inter-domain interaction, between bacteria and fungi, has been associated with inflammatory responses in humans and model organisms (Krüger *et al.* 2019). This type of interaction, however, cannot be found using existing methods that treat each compositional vector separately.

## 2 Methods

### 2.1 Additive logistic normal model for multiple compositional vectors

Assume that there are *d* compositional vectors $X^{1},\ldots ,X^{d}$ observed in microbiome studies that

$$X^{i}={\left(X_{1}^{i},\ldots ,X_{p_{i}}^{i}\right)}^{T},\;$$

and $1_{p_{i}}{}^{T}X^{i}=1$ for 1≤i≤d where *A^T^* is the transpose of a matrix *A* and $1_{m}$ are a m×1 column vector whose elements are all 1s for a positive integer *m*. For the case *d *=* *2 that bacteria and fungi are considered in microbiome data, *p*_1_ and *p*_2_ represent numbers of bacteria and fungi, respectively while *X*^1^ and *X*^2^ are relative abundances for bacteria and fungi, respectively. The relationship between observed compositional vector *X^i^* and the unobserved absolute abundance $Y^{i}={\left(Y_{1}^{i},\ldots ,Y_{p_{i}}^{i}\right)}^{T}$ is

$$X_{j}^{i}=\frac{Y_{j}^{i}}{1_{p_{i}}{}^{T}Y^{i}},\;1\leq j\leq p_{i},\;1\leq i\leq d.\;$$

Let $\ln x={\left(\ln x_{1},\ldots ,\ln x_{m}\right)}^{T}$ for a m×1 column vector $x={\left(x_{1},\ldots ,x_{m}\right)}^{T}$ for a positive integer *m*,

$$\ln X=\left(\begin{array}{c} \ln X^{1}\\ \vdots \\ \ln X^{d} \end{array}\right),\;\ln Y=\left(\begin{array}{c} \ln Y^{1}\\ \vdots \\ \ln Y^{d} \end{array}\right),\;W=\left(\begin{array}{c} \ln(1_{p_{1}}{}^{T}Y^{1})\\ \vdots \\ \ln(1_{p_{d}}{}^{T}Y^{d}) \end{array}\right),\;$$

and

$$R=\left(\begin{array}{cccc} 1_{p_{1}} & 0 & \cdots & 0\\ 0 & \ddots & \ddots & \vdots \\ \vdots & \ddots & \ddots & 0\\ 0 & \cdots & 0 & 1_{p_{d}} \end{array}\right),\;$$

then the relationship between lnX and lnY is

(1) lnY=lnX+RW. 

Assume the absolute abundance lnY is from a multivariate normal distribution with mean *μ* and a precision matrix Ω that

$$\ln Y∼N_{p}(\mu ,\Omega^{-1})$$

where $p=\sum_{i=1}^{d}p_{i}$ is the total number of microbes considered in the analysis. The precision matrix Ω is the inverse of the covariance matrix and defines an undirected network that each node represents one random variable, and two nodes are connected if and only if the corresponding element in Ω is not 0. The goal is to estimate the precision matrix of lnY from its compositional realization lnX. The structure of non-zero elements in Ω reveals the conditional dependence among the latent variables lnY. Since the total absolute abundance *W* is lost for the absolute abundance lnY, the identifiability of Ω cannot be guaranteed without any assumption on Ω. A commonly adopted assumption is that Ω is sparse and has only a small number of edges. The sparse assumption on Ω has two interpretations in practice. First, when the number of observations is small, the observed compositional data may be fitted well by a sparse precision matrix with fewer parameters. Second, only several strongest edges are of interest, and edges with extremely small strengths can be ignored in the network.

### 2.2 Penalized likelihood for Ω

The distribution for (*W*, *X*) can be derived from the transformation (1) that

$$\begin{array}{cc} f_{W,X}(w,x)= & \frac{|\Omega {|}^{\frac{1}{2}}\;\text{exp}\;\left[-\frac{1}{2}{\left(\ln x+Rw-\mu \right)}^{T}\Omega (\ln x+Rw-\mu )\right]}{{\left(2\pi \right)}^{\frac{p}{2}}\prod_{i=1}^{d}\prod_{j=1}^{p_{i}}x_{j}^{i}},\; \end{array}$$

where |A| represents the determinant of a square matrix *A* and the symbol *x* represents

$${\left(x_{1}^{1},\ldots ,x_{p_{1}-1}^{1},\ldots ,x_{1}^{d},\ldots ,x_{p_{d}-1}^{d}\right)}^{T}$$

when it appears on the left of the expression of a distribution function and

$${\left(x_{1}^{1},\ldots ,x_{p_{1}}^{1},\ldots ,x_{1}^{d},\ldots ,x_{p_{d}}^{d}\right)}^{T}$$

when it appears on the right. Then the conditional distribution of *W* given *X* can be derived from the form of $f_{W,X}(w,x)$ that

$$W|X∼N_{d}\left(-{\left(R^{T}\Omega R\right)}^{-1}R^{T}\Omega (\ln X-\mu ),{\left(R^{T}\Omega R\right)}^{-1}\right).\;$$

Let $Q=\Omega -\Omega R{\left(R^{T}\Omega R\right)}^{-1}R^{T}\Omega$, then the marginal distribution of *X* is

$$\begin{array}{c} f_{X}(x)=\frac{f_{W,X}(w,x)}{f_{W|X}(w|x)}=\frac{|\Omega {|}^{\frac{1}{2}}\;\text{exp}\;\left[-\frac{1}{2}{\left(\ln x-\mu \right)}^{T}Q(\ln x-\mu )\right]}{|R^{T}\Omega R{|}^{\frac{1}{2}}\left[{\left(2\pi \right)}^{\frac{p-d}{2}}\prod_{i=1}^{d}\prod_{j=1}^{p_{i}}x_{j}^{i}\right]}.\; \end{array}$$

Let $P=E_{p}-R{\left(R^{T}R\right)}^{-1}R^{T}$ where $E_{p}$ is the *p *×* p* identity matrix, then from *PQP* = *Q*, we have

$$\begin{array}{cc} f_{X}(x) & =\frac{|\Omega {|}^{\frac{1}{2}}\;\text{exp}\;\left[-\frac{1}{2}{\left(P\ln x-P\mu \right)}^{T}Q(P\ln x-P\mu )\right]}{|R^{T}\Omega R{|}^{\frac{1}{2}}\left[{\left(2\pi \right)}^{\frac{p-d}{2}}\prod_{i=1}^{d}\prod_{j=1}^{p_{i}}x_{j}^{i}\right]}.\; \end{array}$$

Let the sample mean of PlnX be the estimator of Pμ and *S* be the sample variance of lnX for independent and identically distributed samples of *X*. Then the negative log-likelihood of Ω is

(2) $$\begin{array}{c} L(\Omega )=-\ln|\Omega |+\ln|R^{T}\Omega R|\\ +Tr\left(S[\Omega -\Omega R{\left(R^{T}\Omega R\right)}^{-1}R^{T}\Omega ]\right)\; \end{array}$$

up to a constant not depending on Ω where Tr(A) is the trace of a square matrix *A*. The derivation of the likelihood of Ω is a natural extension for the case with single compositional vector (Fang *et al.* 2017). Under the sparse assumption of Ω, the following estimator, named gmcoda, is considered for inferring the precision matrix Ω,

(3) $$\hat{\Omega }=\underset{\Omega ≻0}{\arg\min}f(\Omega )=\underset{\Omega ≻0}{\arg\min}L(\Omega )+\lambda ||\Omega |{|}_{1},\;$$

where

(4) $$f(\Omega )=L(\Omega )+\lambda ||\Omega |{|}_{1}$$

is the objective function, $\left||\Omega |{|}_{1}=\sum_{1\leq i,j\leq p}|\Omega_{ij}\right|$ and Ω≻0 means Ω is positive definite. The tuning parameter *λ* is selected via the Bayesian information criteria. The estimation of the partial correlation matrix can be derived by standardizing $\hat{\Omega }$.

### 2.3 Optimization algorithm

A majorization–minimization algorithm can be used to solve the optimization problem in the penalized likelihood in Equation (3). Let

$$\begin{array}{c} S_{\Omega }=\left[E_{p}-R{\left(R^{T}\Omega R\right)}^{-1}R^{T}\Omega ]S[E_{p}-\Omega R{\left(R^{T}\Omega R\right)}^{-1}R^{T}\right]\\ +R{\left(R^{T}\Omega R\right)}^{-1}R^{T},\; \end{array}$$

The negative log-likelihood of Ω in Equation (2) can be rewritten as

$$\begin{array}{c} L(\Omega )=-\ln\frac{\left|\Omega \right|}{\left|R^{T}\Omega R\right|}+Tr\left(\left[S_{\Omega }-R{\left(R^{T}\Omega R\right)}^{-1}R^{T}\right]\Omega \right).\; \end{array}$$

Let Ω_*k*_ be the value of Ω in the *k*th step in an iteration algorithm, then the following function $g(\Omega |\Omega_{k})$ is a majorization function for the objective function f(Ω) in Equation (4),

(5) $$g(\Omega |\Omega_{k})=-\ln|\Omega |+Tr\left(\Omega S_{\Omega_{k}}\right)+\lambda ||\Omega |{|}_{1}+\ln|R^{T}\Omega_{k}R|-d,\;$$

that $g(\Omega |\Omega_{k})$ satisfies the following two properties,

$$\begin{array}{cc} & g(\Omega_{k}|\Omega_{k})=f(\Omega_{k}),\;\\ & g(\Omega |\Omega_{k})\geq f(\Omega ).\; \end{array}$$

It’s trivial that $g(\Omega_{k}|\Omega_{k})=f(\Omega_{k})$. We give a proof for $g(\Omega |\Omega_{k})\geq f(\Omega )$ as follows. Let $A^{⊗2}=AA^{T}$ for a matrix *A* and $S^{\frac{1}{2}}$ be the positive semi-definite square root of *S*, then

$$\begin{array}{c} g(\Omega |\Omega_{k})-f(\Omega )\\ =Tr\left(\Omega [S_{\Omega_{k}}-R{\left(R^{T}\Omega_{k}R\right)}^{-1}R^{T}]-S[\Omega -\Omega R{\left(R^{T}\Omega R\right)}^{-1}R^{T}\Omega ]\right)\\ +\ln|R^{T}\Omega_{k}R|-\ln|R^{T}\Omega R|+Tr\left(R{\left(R^{T}\Omega_{k}R\right)}^{-1}R^{T}(\Omega -\Omega_{k})\right)\\ =Tr\left((R^{T}\Omega R){\left[{\left(R^{T}\Omega R\right)}^{-1}R^{T}\Omega S^{\frac{1}{2}}-{\left(R^{T}\Omega_{k}R\right)}^{-1}R^{T}\Omega_{k}S^{\frac{1}{2}}\right]}^{⊗2}\right)\\ +\ln|R^{T}\Omega_{k}R|+Tr\left(R{\left(R^{T}\Omega_{k}R\right)}^{-1}R^{T}(\Omega -\Omega_{k})\right)-\ln|R^{T}\Omega R|\\ \geq \ln|R^{T}\Omega_{k}R|+Tr\left(R{\left(R^{T}\Omega_{k}R\right)}^{-1}R^{T}(\Omega -\Omega_{k})\right)-\ln|R^{T}\Omega R|.\; \end{array}$$

Let $f_{1}(\Omega )=-\ln|R^{T}\Omega R|$ and d be the differential operator, then

$$\begin{array}{c} df_{1}(\Omega )=-Tr\left(\left[R{\left(R^{T}\Omega R\right)}^{-1}R^{T}\right]d\Omega \right),\\ d^{2}f_{1}(\Omega )=Tr\left(\left[R{\left(R^{T}\Omega R\right)}^{-1}R^{T}\right]d\Omega \left[R{\left(R^{T}\Omega R\right)}^{-1}R^{T}\right]d\Omega \right).\; \end{array}$$

So $f_{1}(\Omega )$ is a convex function with respect to Ω and

$$\begin{array}{c} f_{1}(\Omega )-f_{1}(\Omega_{k})=-\ln|R^{T}\Omega R|+\ln|R^{T}\Omega_{k}R|\\ \geq -Tr\left(R{\left(R^{T}\Omega_{k}R\right)}^{-1}R^{T}(\Omega -\Omega_{k})\right).\; \end{array}$$

Then

$$\begin{array}{ccc} & g(\Omega |\Omega_{k})-f(\Omega ) & \geq 0.\; \end{array}$$

So $g(\Omega |\Omega_{k})$ is a majorization function for f(Ω) and we can minimize f(Ω) by solving a series of optimization problem as follows,

(6) $$\Omega_{k+1}=\underset{\Omega ≻0}{\arg\min}g(\Omega |\Omega_{k})=\underset{\Omega ≻0}{\arg\min}-\ln|\Omega |+Tr\left(\Omega S_{\Omega_{k}}\right)+\lambda ||\Omega |{|}_{1},\;$$

for k=0,1,2,… until Ω_*k*_ converges. The optimization problem in Equation (6) can be solved by the graphical lasso algorithm (Friedman *et al.* 2008).

## 3 Results

### 3.1 Simulation studies

Simulation studies are performed to compare gmcoda with other methods for estimating the partial correlation matrix. Graphical lasso with logarithm transformation of proportions is denoted as glasso. SPIEC-EASI variants with sparse neighborhood and covariance selection are denoted as SEMB and SEGL, respectively. In simulations, each element of the mean vector for latent variables lnY is independently generated from the uniform distribution on [−0.5,0.5]. The precision matrix Ω is generated in the following way. A predefined network is selected to decide the network structure. Simulation studies consider the following six structures: random, neighbor, band, hub, block, and scale-free networks, as described below.

- Random network: Pairs of nodes are connected with probability 0.1 and the strengths of edges are set 0.15 and −0.15 with equal probabilities.
- Neighbor network: A distance matrix of *p* nodes is first constructed based on the random positions on ${\left[0,1\right]}^{2}$ and each node is connected with its eight closest neighbors. The strengths of edges are set as 0.15 and −0.15 with equal probabilities.
- Band network: Nodes are sorted in a linear order and each node is connected with its five closest neighbors. The strengths of edges are set 0.4, 0.2, −0.2, −0.1, and −0.1 as the distances are 1, 2, 3, 4, and 5.
- Hub network: Four nodes are selected as hub nodes. Hub nodes are connected with hub nodes with probability 1 and connected with non-hub nodes with probability 0.75. The strength of edges involved with hub nodes is set as 0.1. Pairs of non-hub nodes are connected with probability 0.05 and strength 0.15.
- Block network: Nodes are divided into eight blocks with nearly equal sizes. Pairs of nodes in the same block are connected with probability 0.45 and strength 0.2. Pairs of nodes from different blocks are connected with probability 0.05 and strength 0.15.
- Scale-free network: The Barabási–Albert model (Barabási and Albert 1999) is used with a power coefficient of 2 and 10 edges are added in each step. The strengths of edges are generated from a uniform distribution on a discrete set {−0.8,−0.5,0.5,0.8}.

Let *p* be the number of nodes. The edge densities for random, neighbor, band, hub, block, and scale-free networks are about 0.1, 12/p, 10/p, 0.05+6/p, 0.1, and 10/p, respectively. The number of nodes is set as *p* = 50 and the sample size is set as *n* = 100 and *n* = 300. The diagonal elements of the precision matrix are set large enough to make the matrix positive definite. Two compositional vectors (*d* = 2) are considered in simulation studies that $\left(p_{1},p_{2})=(25,25\right)$ and $\left(p_{1},p_{2})=(30,20\right)$.

We first compare gmcoda with gcoda (Fang *et al.* 2017) in recovering edges from compositional data since gmcoda can be seen as an extension for multiple compositional vectors of gcoda. Two scenarios are considered: First, two compositional vectors are sub-components from the single compositional vector; Second, two compositional vectors are first generated and then directly merged into a single compositional vector. The performances of gmcoda and gcoda are similar in the first scenario while gmcoda performs better than gcoda for most cases in the second scenario (Supplementary Table S1). The overlap of shared edges between gmcoda and gcoda in the first scenario is higher than that in the second scenario. The results of these two approaches are consistent if multiple compositional vectors are sub-components of one compositional vector.

Table 1 is the performance comparison of the inferred partial correlation matrix from gmcoda, glasso, and SPIEC-EASI with $\left(n,p_{1},p_{2})=(300,25,25\right)$. The precision matrix for glasso is estimated from the stable selection like that in SPIEC-EASI. Table 1 uses the following six measures for comparing these methods: false positive rate (FPR), true positive rate (TPR, also called recall), precision, F1 score and two accuracy measures for estimation,

$$\begin{array}{cc} d_{1} & =\frac{1}{p(p-1)}\sum_{1\leq i\neq j\leq p}|{\hat{\rho }}_{ij}-\rho_{ij}|,\;\\ d_{f} & =\sqrt{\frac{1}{p(p-1)}\sum_{1\leq i\neq j\leq p}{\left({\hat{\rho }}_{ij}-\rho_{ij}\right)}^{2}},\; \end{array}$$

where $\hat{\rho }$ is the estimation of the true partial correlation matrix *ρ*. Last column of Table 1 is the running time for the compared four methods under a Linux workstation: Intel(R) Xeon(R) E7-4820 v4 (2.00 GHz) CPU and 251 GB MEM. FPR and TPR of glasso and SPIEC-EASI are very small that these methods would return much more sparse estimations than gmcoda. Gmcoda performs better than its competitors via F1 scores. The errors *d*_1_ and $d_{f}$ for gmcoda are also the lowest among the compared four methods. Gmcoda is the fastest algorithm among these four algorithms. The case that the number of microbes is no less than the sample size is also considered in simulation studies (Supplementary Tables S2 and S3). The result of p≥n is similar to the case of *p *<* n*.

**Table 1.** Performance comparison of gmcoda, glasso and SPIEC-EASI for the inferred partial correlation matrix in simulation studies with $\left(n,p_{1},p_{2})=(300,25,25\right)$.^a^

Network	Method	FPR	TPR (Recall)	Precision	F1 score	*d*_1_	$d_{f}$	Time (s)
Random	gmcoda	0.023	0.592	0.768	0.651	0.012	0.039	0.242
	glasso	0.000	0.013	0.148	0.023	0.015	0.047	25.971
	SEGL	0.000	0.016	0.546	0.029	0.015	0.047	38.180
	SEMB	0.000	0.016	0.546	0.029	–	–	37.196
Neighbor	gmcoda	0.084	0.739	0.676	0.704	0.019	0.046	0.357
	glasso	0.001	0.120	0.980	0.212	0.027	0.063	25.173
	SEGL	0.001	0.117	0.976	0.209	0.027	0.063	38.925
	SEMB	0.000	0.089	0.990	0.162	–	–	34.560
Band	gmcoda	0.151	0.661	0.511	0.576	0.022	0.051	0.468
	glasso	0.000	0.267	1.000	0.421	0.036	0.096	24.726
	SEGL	0.000	0.339	0.850	0.476	0.035	0.091	39.681
	SEMB	0.000	0.368	0.749	0.469	–	–	35.569
Hub	gmcoda	0.014	0.148	0.646	0.233	0.017	0.046	0.238
	glasso	0.002	0.024	0.607	0.046	0.017	0.045	25.283
	SEGL	0.002	0.030	0.618	0.056	0.017	0.045	36.890
	SEMB	0.002	0.028	0.614	0.053	–	–	36.293
Block	gmcoda	0.022	0.514	0.740	0.574	0.015	0.049	0.262
	glasso	0.000	0.015	0.250	0.027	0.017	0.056	23.923
	SEGL	0.000	0.052	0.550	0.084	0.017	0.055	36.163
	SEMB	0.000	0.063	0.548	0.102	–	–	34.464
Scale-free	gmcoda	0.169	0.884	0.540	0.666	0.017	0.040	0.502
	glasso	0.006	0.119	0.701	0.203	0.026	0.062	26.543
	SEGL	0.006	0.116	0.762	0.193	0.026	0.062	43.301
	SEMB	0.004	0.134	0.893	0.232	–	–	36.964

a SEMB and SEGL are two options for SPIEC-EASI. FPR and TPR (also called recall) are the false positive rate and the true positive rate, respectively. *d*_1_ and $d_{f}$ for SEMB are not available since SEMB doesn’t return an estimated precision matrix. The results are the averages over 20 runs.

A noticeable phenomenon in Table 1 is the moderate–low recalls in most cases. The recall of gmcoda is larger than that of other methods in all cases. One possible reason is that glasso, SEGL, and SEMB are more conservative than gmcoda. Glasso, SEGL, and SEMB produced fewer edges than gmcoda but did not get significant improvements in precision for recovering networks in some cases. It seems the identification efficiency of interactions depends on the strength of partial correlation coefficients in the precision matrix. We compare the identification efficiency of edges grouped by the strength of partial correlation coefficients in the band network (Supplementary Fig. S1). We find that the proportions of identified edges for gmcoda are larger than other methods in all four non-zeros partial correlation coefficients and there is an obvious trend that the stronger the strengths of the underlying true edges, the more likely detected by gmcoda. This moderate–low recall phenomenon reminds researchers that current methods may miss some interesting edges among microbes for interpreting microbial networks.

We also compare the area under the curve (AUC) values of various combinations of the network structure, the dimension of microbes, and the sample size (Supplementary Table S4). AUC values are calculated by varying the tuning parameter. AUC values are used as the secondary measure for comparison since AUC values do not provide useful information about the estimation that will be used in practice. Both sample sizes and lengths of composition vectors influence the performance of all methods. As the sample size increases, AUC values increase for all methods. The performance of gmcoda is much better than glasso and SPEIC-EASI for recovering the network structure of random, neighbor, and scale-free networks via AUC values. For hub and block networks, SPIEC-EASI performs better than gmcoda and glasso. For the band network, glasso is the best for a large sample size (*n *=* *300) while SPIEC-EASI is the best for a small sample size (*n *=* *100).

We also conducted a simulation study for comparing gmcoda and other methods in a situation where there are only inter-domain interactions while no cross-domain correlation exists. Compared to gmcoda’s results that about 15% of detected cross-domain edges, the other methods almost detect no false edges for bacteria–fungi interaction (Supplementary Table S5). However, gmcoda showed the advantage of the recall and the precision in this scenario.

### 3.2 Application in microbiome studies

We applied the proposed approach to infer the interaction network among bacteria and fungi in an intestinal bacterial microbiome dataset from fecal samples (Haak *et al.* 2021). This data contains 59 samples that are subjected to 16S rRNA genes with V3–V4 regions for bacteria and ITS rDNA gene sequences for fungi. The data are filtered by removing operational taxonomic units (OTUs) with more than 80% 0s across samples and removing samples with more than 80% 0s across OTUs separately for bacteria and fungi. There are 33 bacteria OTU and 15 fungi OTU remaining for 47 samples after the filtering process.

**Figure 1.**
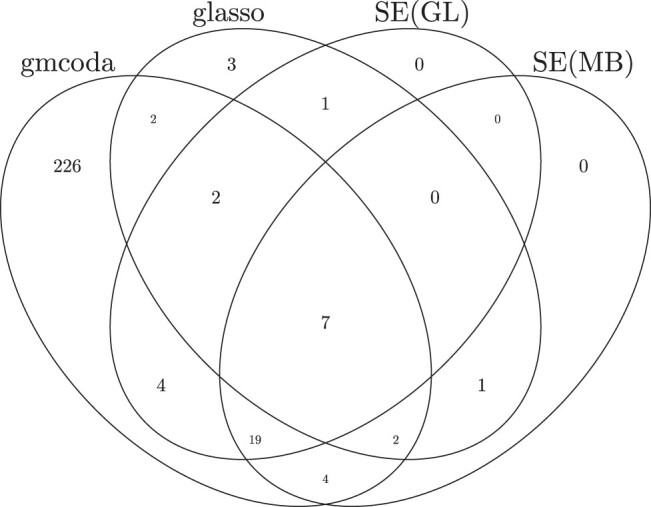
Venn diagrams of shared edges for networks inferred from gmcoda, glasso, and SPIEC-EASI for the intestinal bacterial microbiome data.

The counts of edges inferred by gmcoda, glasso, and SPIEC-EASI are presented in Table 2. The inferred network from gmcoda includes 266 edges, which are substantially more than those from glasso and SPIEC-EASI. The overlapped edges among these four methods are shown in Fig. 1. Most edges inferred by glasso and SPIEC-EASI are also detected by gmcoda. We shuffle the count data by permuting the counts in each OTU to give an estimate of the false discovery count. The permutation is repeated 20 times for deriving a reliable estimation of the false discovery count. The false discovery counts of gmcoda, glasso, SEMB, and SEGL are 10.30, 6.70, 7.60, and 7.25, respectively. The false discovery count of gmcoda is comparable to that of its competitors.

**Table 2.** Edge numbers for networks inferred by gmcoda, glasso, and SPIEC-EASI for the intestinal bacterial microbiome data.

Method	Bacteria–Bacteria	Bacteria–Fungi	Fungi–Fungi	Total
gmcoda	148	81	37	266
glasso	11	0	7	18
SEGL	23	1	9	33
SEMB	25	1	7	33

The network inferred by gmcoda includes 81 connections between bacteria and fungi. The strongest inter-domain edge connects the bacterial genus in *Agathobacter* and the fungal genus in *Aspergillus*, with an estimated partial correlation of −0.21; SPIEC-EASI also estimated a significant, albeit weaker, partial correlation of −0.028. Consistent with this finding, an independent gut microbiome study in COVID-19 patients has reported a negative correlation between *Aspergillus niger* and *Agathobacter* (Lv *et al.* 2021). A second strongest inter-domain edge, detected only by gmcoda and not by SPIEC-EASI, indicates a positive partial correlation between bacterial genus *Streptococcus* and fungal genus *Candida*. This finding is supported by an independent dental microbiome study, which has reported a statistically significant, and positive, correlation between *Candida albicans* and *Streptococcus mutans* (Sridhar *et al.* 2020).

## 4 Discussion

The interactions between bacteria and fungi are related to human health and disease (Krüger *et al.* 2019). We propose a graphical model approach, called gmcoda, for inferring microbial cross-domain interactions in microbiome studies. The proposed model is based on a latent logistic normal model for compositional data in that multiple compositional vectors are considered simultaneously. An efficient algorithm is developed to solve the optimization problem involved in gmcoda. Simulation studies show that gmcoda provides more accurate and robust results than SPIEC-EASI and glasso in most cases. Gmcoda provides larger AUCs than SPIEC-EASI for scale-free networks in simulation studies. Gmcoda is also applied in the network inference of real microbiome data and can find connections between fungi and bacteria, which would not be possible when using existing methods that treat each compositional vector separately.

Although gmcoda provides a novel method for inferring microbial cross-domain interactions, there are still some challenges in the field of network inference from microbiome data. The first challenge is that almost all methods, including all methods mentioned in this paper, based on log-ratio transformations assume the observed zeros are not true zeros and these observed zeros should be imputed with some small positive number before calculating compositions. This assumption is not always true for complex microbial ecosystems which some microbes don’t live in some conditions. More reasonable statistical models should be built for considering true zeros in compositional data. One strategy for this zero-value problem is using a mixture model of the single point distribution at zero and the additive logistical normal distribution instead of a single logistical normal distribution. The second challenge is the selection of the tuning parameter for balancing the model fitting and the sparsity assumption in gmcoda. Currently, gmcoda applied Bayesian information criteria for choosing the tuning parameter and simulation results showed some false edges are involved in the estimation. One strategy for choosing the tuning parameter is the adaptive method with two stages. The third challenge is the computational complexity of large-scale datasets in inferring networks. Although gmcoda is much faster than other methods, gmcoda is time-consuming when the number of microbes is moderate–large. A potential future direction for inferring microbial networks is to develop scalable algorithms for dealing with large datasets in real-world scenarios.

## Supplementary Material

btad700_Supplementary_Data

## Data Availability

The data underlying this article are available in the article and in its online supplementary material.
