# Supplementary material for: gmcoda: Graphical model for multiple compositional vectors in microbiome studies
Source: Bioinformatics. 2023 Nov 17;39(11):btad700. doi: 10.1093/bioinformatics/btad700 (PMC10681862; doi:10.1093/bioinformatics/btad700)
Supplement: btad700_Supplementary_Data [file btad700_supplementary_data.pdf]

Supplementary Material for "gmcoda: Graphical model for multiple compositional vectors in microbiome studies"

Huaying Fang[1,2]

[1]Beijing Advanced Innovation Center for Imaging Theory and Technology, Capital Normal University, Beijing 100048, China.

[2]Academy of Multidisciplinary Studies, Capital Normal University, Beijing 100048, China.

1. Simulation settings for comparing gmcoda and gcoda in recovering edges from compositional data

The sample size is 300, the numbers of two sub-compositional vectors are both set as 25, network structures are described in the main text and all simulations are replicated 20 times for calculating averages. This simulation study includes two scenarios. For the first scenario, the single compositional vector with a length of 50 is first generated from the additive logistical normal distribution, and then two sub-compositional vectors each with a length of 25 are derived as sub-components from the single compositional vector. For the second scenario, two compositional vectors each with a length of 25 are first generated, and then a single compositional vector is calculated by merging two compositional vectors into one and normalizing to get a compositional vector.

2. Simulation settings for no cross-domain interactions

The sample size is 300, the numbers of bacteria and fungi vectors are both set as 25, and network structures for bacteria-bacteria or fungi-fungi are set as the same where there is no edge between bacteria and fungi. All simulations are replicated 20 times to calculate the averages.

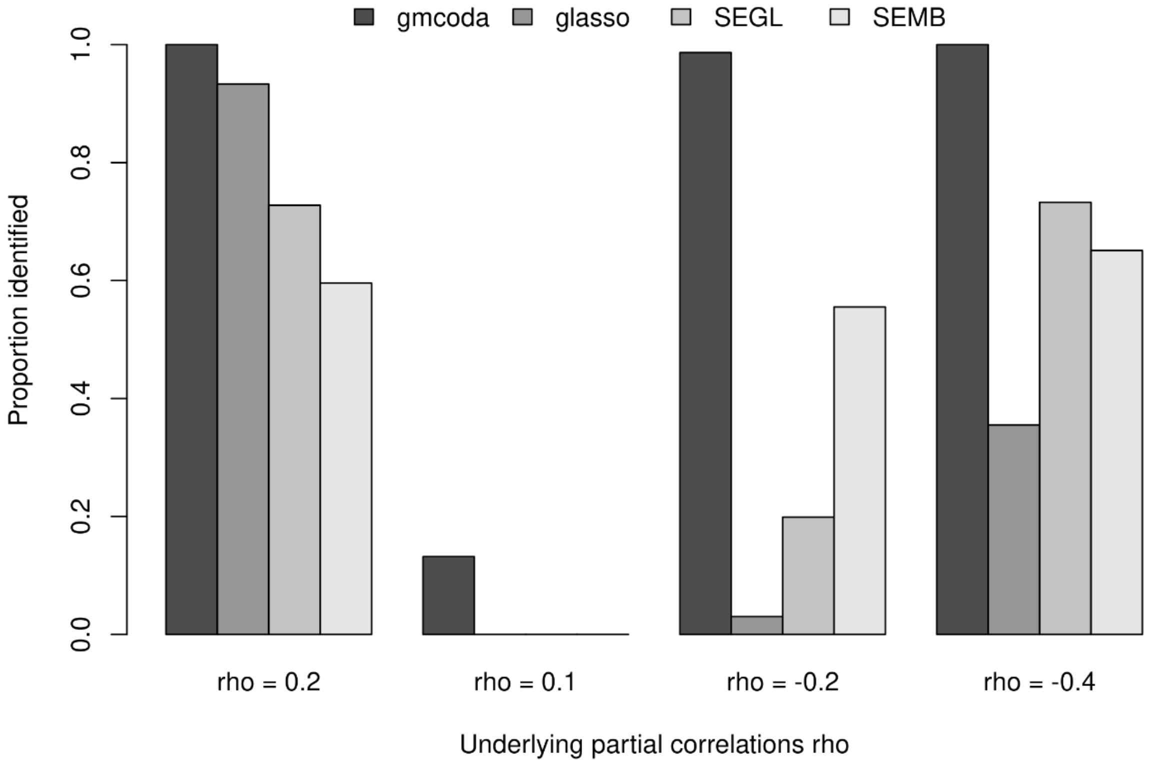

Fig. S1. Proportion of identified edges stratified by the underlying partial correlation coefficients for the band network from Table 1 in the main text. The results are the averages over 20 replicates.

Table S1. Simulation results of applying gcoda and gmcoda for compositional vectors. The results are the averages over 20 replicates. The measure “Overlap” is defined as the ratio of the number of shared edges and the minimum number of edges detected by gmcoda or gcoda.

			Network					
Scenario			Random	Neighbor	Band	Hub	Block	Scale-free
1	F1 Score	gmcoda	0.65	0.70	0.58	0.26	0.56	0.68
		gcoda	0.68	0.71	0.58	0.26	0.64	0.69
	Overlap		0.96	0.95	0.94	0.95	0.96	0.89
2	F1 Score	gmcoda	0.66	0.70	0.59	0.28	0.57	0.68
		gcoda	0.64	0.66	0.61	0.28	0.58	0.67
	Overlap		0.94	0.90	0.92	0.90	0.97	0.88

Table S2. Performance comparison of gmcoda, glasso and SPIEC-EASI for the inferred partial correlation matrix in simulation studies with $(n, p_1, p_2) = (100, 50, 50)$. The results are the averages over 20 runs.

Network	Method	FPR	TPR (Recall)	Precision	F1 Score	d_1	d_f	Time (s)
Random	gmcoda	0.014	0.182	0.626	0.275	0.015	0.047	22.559
	glasso	0.005	0.108	0.699	0.185	0.015	0.048	101.21
	SEGL	0.005	0.114	0.755	0.194	0.015	0.048	108
	SEMB	0.003	0.099	0.8	0.174	-	-	89.844
Neighbor	gmcoda	0.005	0.164	0.827	0.267	0.014	0.044	19.053
	glasso	0.002	0.115	0.825	0.2	0.014	0.045	97.205
	SEGL	0.002	0.13	0.88	0.223	0.014	0.045	103.51
	SEMB	0.002	0.116	0.885	0.203	-	-	88.04
Band	gmcoda	0.015	0.409	0.759	0.529	0.017	0.061	23.581
	glasso	0.001	0.205	0.974	0.338	0.019	0.069	95.177
	SEGL	0.001	0.253	0.983	0.401	0.019	0.067	102.1
	SEMB	0	0.235	0.989	0.378	-	-	89.362
Hub	gmcoda	0.017	0.205	0.588	0.301	0.011	0.035	15.528
	glasso	0.001	0.006	0.138	0.012	0.011	0.033	882.27
	SEGL	0.009	0.156	0.666	0.251	0.01	0.034	105.8
	SEMB	0.005	0.085	0.653	0.15	-	-	80.757
Block	gmcoda	0.019	0.194	0.548	0.27	0.017	0.053	23.61
	glasso	0.006	0.081	0.383	0.132	0.017	0.055	100.27
	SEGL	0.006	0.157	0.728	0.252	0.017	0.054	105.02
	SEMB	0.004	0.159	0.817	0.263	-	-	89.832
Scale-free	gmcoda	0.01	0.192	0.681	0.295	0.01	0.033	21.83
	glasso	0.003	0.08	0.737	0.143	0.01	0.034	99.511
	SEGL	0.003	0.085	0.783	0.152	0.01	0.034	107.96
	SEMB	0.002	0.076	0.817	0.137	-	-	87.434

Table S3. Performance comparison of gmcoda, glasso and SPIEC-EASI for the inferred partial correlation matrix in simulation studies with $(n, p_1, p_2) = (80, 50, 50)$. The results are the averages over 20 runs.

Network	Method	FPR	TPR (Recall)	Precision	F1 Score	d_1	d_f	Time (s)
Random	gmcoda	0.012	0.147	0.616	0.229	0.015	0.047	71.221
	glasso	0.003	0.051	0.41	0.09	0.016	0.048	128.21
	SEGL	0.004	0.07	0.491	0.12	0.016	0.048	133.47
	SEMB	0.004	0.091	0.709	0.16	-	-	66.078
Neighbor	gmcoda	0.005	0.113	0.795	0.185	0.014	0.045	63.512
	glasso	0.002	0.083	0.575	0.143	0.014	0.046	124.58
	SEGL	0.003	0.107	0.846	0.183	0.014	0.045	128.48
	SEMB	0.002	0.103	0.847	0.179	-	-	64.008
Band	gmcoda	0.015	0.316	0.76	0.423	0.018	0.064	50.27
	glasso	0.001	0.186	0.95	0.309	0.019	0.069	116.21
	SEGL	0.001	0.22	0.962	0.355	0.019	0.068	122.33
	SEMB	0.001	0.201	0.97	0.331	-	-	65.229
Hub	gmcoda	0.017	0.189	0.565	0.281	0.011	0.035	30.117
	glassi	0.002	0.006	0.092	0.011	0.011	0.033	834.05
	SEGL	0.006	0.082	0.611	0.134	0.011	0.034	123.63
	SEMB	0.008	0.089	0.572	0.153	-	-	58.988
Block	gm	0.012	0.12	0.562	0.185	0.017	0.054	61.957
	glasso	0.005	0.052	0.273	0.086	0.017	0.055	123.75
	SEGL	0.005	0.105	0.617	0.174	0.017	0.055	127.32
	SEMB	0.004	0.127	0.723	0.213	-	-	64.291
Scale-free	gmcoda	0.01	0.171	0.649	0.266	0.01	0.034	90.683
	glasso	0.005	0.078	0.658	0.138	0.011	0.034	123.31
	SEGL	0.004	0.077	0.705	0.137	0.01	0.034	128.81
	SEMB	0.003	0.064	0.734	0.116	-	-	62.689

Table S4. Performance comparison of gmcoda, glasso and SPIEC-EASI via the area under the curve (AUC) values for recovering network structures in simulation studies. The AUC value is the area under the receiver operating characteristic curve (ROC) while ROC is obtained by varying tuning parameters. The results are the averages over 20 runs.

		Network Structure					
(n, p_1, p_2)	Method	Random	Neighbor	Band	Hub	Block	Scale-free
(100,25,25)	gmcoda	0.753	0.772	0.767	0.622	0.746	0.794
	glasso	0.74	0.768	0.755	0.609	0.743	0.754
	SEGL	0.733	0.763	0.773	0.631	0.82	0.752
	SEMB	0.702	0.727	0.747	0.618	0.825	0.725
(100,30,20)	gmcoda	0.757	0.772	0.769	0.616	0.745	0.802
	glasso	0.745	0.767	0.756	0.607	0.745	0.76
	SEGL	0.737	0.763	0.772	0.628	0.828	0.757
	SEMB	0.707	0.729	0.748	0.612	0.834	0.732
(300,25,25)	gmcoda	0.93	0.912	0.834	0.709	0.926	0.916
	glasso	0.912	0.887	0.845	0.726	0.91	0.87
	SEGL	0.879	0.869	0.791	0.749	0.958	0.855
	SEMB	0.851	0.848	0.779	0.734	0.958	0.844
(300,30,20)	gmcoda	0.934	0.911	0.835	0.707	0.925	0.922
	glasso	0.913	0.885	0.847	0.725	0.913	0.874
	SEGL	0.886	0.871	0.796	0.749	0.964	0.863
	SEMB	0.853	0.846	0.781	0.737	0.966	0.851

Table S5. Simulation results for no interaction exists between bacteria and fungi. The results are the averages over 20 replicates. The measure “Intra-Error” is defined as the proportion of false bacteria-fungi edges among all detected edges for a given algorithm.

		Network					
Method	Measure	Random	Neighbor	Band	Hub	Block	Scale-free
gmcoda	Intra-Error	0.08	0.16	0.29	0.12	0.14	0.10
	Recall	0.36	0.80	0.67	0.06	0.62	0.70
	Precision	0.81	0.64	0.52	0.38	0.64	0.74
glasso	Intra-Error	0.00	0.00	0.00	0.01	0.00	0.00
	Recall	0.08	0.21	0.37	0.00	0.23	0.09
	Precision	0.62	0.98	1.00	0.08	0.97	0.84
SEGL	Intra-Error	0.00	0.00	0.00	0.01	0.00	0.00
	Recall	0.07	0.41	0.32	0.02	0.35	0.08
	Precision	0.74	0.93	0.85	0.58	1.00	0.84
SEMB	Intra-Error	0.00	0.00	0.00	0.01	0.00	0.00
	Recall	0.07	0.25	0.41	0.02	0.35	0.08
	Precision	0.74	0.99	0.90	0.58	1.00	0.89
